# Full-length cytokeratin-19 is released by human tumor cells: a potential role in metastatic progression of breast cancer

**DOI:** 10.1186/bcr2326

**Published:** 2009-06-23

**Authors:** Catherine Alix-Panabières, Jean-Pierre Vendrell, Monique Slijper, Olivier Pellé, Eric Barbotte, Grégoire Mercier, William Jacot, Michel Fabbro, Klaus Pantel

**Affiliations:** 1 Department of Virology, Lapeyronie Hospital, University Medical Center of Montpellier, 371, avenue du Doyen Gaston Giraud, 34295 Montpellier Cedex 5, France; 2 INSERM U847, Biotherapy of Normal and Cancer Stem Cells, 99, rue Puech Villa. 34295 Montpellier, Cedex 5, France; 3 Department of Biomolecular Mass Spectrometry, Bijvoet Center for Biomolecular Research and Utrecht Institute for Pharmaceutical Sciences, Utrecht University, Sorbonnelaan 16, 3584 CA Utrecht, The Netherlands; 4 Medical Information Department, University Medical Center of Montpellier, 371, avenue du Doyen Gaston Giraud, 34295 Montpellier, Cedex 5, France; 5 Val d'Aurelle Clinic, 208, rue des Apothicaires, 34000 Montpellier, France; 6 Institute of Tumor Biology, University Medical Center Hamburg-Eppendorf, Martinistr. 52, D-20246 Hamburg, Germany

## Abstract

**Introduction:**

We evaluated whether CK19, one of the main cytoskeleton proteins of epithelial cells, is released as full-length protein from viable tumor cells and whether this property is relevant for metastatic progression in breast cancer patients.

**Methods:**

EPISPOT (EPithelial ImmunoSPOT) assays were performed to analyze the release of full-length CK19 by carcinoma cells of various origins, and the sequence of CK19 was analyzed with mass spectrometry. Additional functional experiments with cycloheximide, Brefeldin A, or vincristine were done to analyze the biology of the CK19-release. CK19-EPISPOT was used to detect disseminated tumor cells in bone marrow (BM) of 45 breast cancer patients who were then followed up over a median of 6 years.

**Results:**

CK19 was expressed and released by colorectal (HT-29, HCT116, Caco-2) and breast (MCF-7, SKBR3, and MDA-MB-231) cancer cell lines. The CK19-EPISPOT was more sensitive than the CK19-ELISA. Dual fluorescent EPISPOT with antibodies against different CK19 epitopes showed the release of the full-length CK19, which was confirmed by mass spectrometry. Functional experiments indicated that CK19 release was an active process and not simply the consequence of cell death. CK19-releasing cells (RCs) were detectable in BM of 44% to 70% of breast cancer patients. This incidence and the number of CK19-RCs were correlated to the presence of overt metastases, and patients with CK19-RCs had a reduced survival as compared with patients without these cells (*P *= 0.025, log-rank test; *P *= 0.0019, hazard ratio, 4.7; multivariate analysis).

**Conclusions:**

Full-length CK19 is released by viable epithelial tumor cells, and CK19-RCs might constitute a biologically active subset of breast cancer cells with high metastatic properties.

## Introduction

Cytokeratins (CKs) constitute the largest intermediate filament protein subgroup and represent a multigene family with more than 20 different types of polypeptides that are divided into acidic type I (CK9-CK20) and basic type II (CK1-CK8) keratins [1]. Cells of normal epithelium express at least one type I and one type II keratin. CKs form the cytoskeleton of epithelial cells, and their main function is to maintain the epithelial cell integrity. However, other functions include roles in cell signaling, stress responses, and apoptosis [2]. CKs undergo a complex regulation involving post-translational modifications and interactions with self and with various classes of associated proteins [2]. During apoptosis, epithelial cells are targets of caspase-mediated proteolysis [3]. CK19, one of the three main keratins besides CK8 and CK18 expressed in simple or stratified epithelium and in various carcinomas including breast cancer [4], is cleaved by caspase 3, and the soluble fragments are released and detected in cancer patients [5]. In contrast, the release of intact, nondegraded CK molecules has not yet been demonstrated.

Bone marrow (BM) is the common homing site for disseminated tumor cells (DTCs) in patients with epithelial tumors and the predominant site of overt metastasis in breast cancer [6]. However, the survival analysis of large cohorts of breast cancer patients [7] and further immunophenotyping of DTCs has indicated that only a subset of DTCs might be relevant for metastatic relapse [6]. Thus, the characterization of the biologic properties of DTCs is of utmost importance. Interestingly, CK19 expression is the most common single marker used for the RT-PCR-based detection of DTCs in the BM of breast cancer patients, and the detection of DTCs expressing this particular cytokeratin is correlated to an unfavorable prognosis because of metastatic relapse [6]. It can therefore be speculated that CK19 not only is a marker for epithelial tumor cells but also may have some biologically relevant functions in early metastatic spread. In view of the recent hypothesis on the role of cancer stem cells in metastatic spread [6], it also is interesting that CK19 has been suggested as a potential breast stem/progenitor cell marker [8-10]. Thus, it can be speculated that CK19-positive tumor cells might be an important subset of breast cancer cells.

The present investigation is an extension of our initial work [11], including now as novel findings (a) the proof that full-length CK19 is released by cancer cells, (b) detailed clinical data on the cancer patients, and (c) most important, clinical follow-up information including multivariate analysis. Our data suggest that the presence of CK19-releasing tumor cells in BM of breast cancer patients is linked to metastatic progression. This discovery may contribute to a better understanding of the heterogeneous biologic potential of DTCs in breast cancer patients.

## Materials and methods

### Cell lines

Colorectal (HT-29-ATCC: HTB-38; CaCo-2-ATCC: HTB-37; HCT116-ATCC: CCL-247) and mammary (MCF-7-ATCC: HTB-22; SKBR3-ATCC: HTB-30; MDA-MB-231-ATCC: TB-26) adenocarcinoma cell lines were used for the optimization of the CK19-EPISPOT. Head and neck squamous cancer cell lines (SCC-14C/SCC-22A) were kindly provided by Prof. Brakenhoff (Department Otolaryngology, Amsterdam, The Netherlands). ML-1 and C643 thyroïd cancer cell lines were kindly provided by Dr. Grimm (Department of Nuclear Medicine, Regensburg University, Germany) and Dr. Heldin (Department of Genetics and Pathology, Uppsala University Hospital, Uppsala, Sweden), respectively.

HT-29 and HCT 116 cells were maintained in McCoy's 5A medium (Invitrogen, Leiden, The Netherlands); CaCo-2 and C643 cells, in minimal essential medium (Eurobio); and MCF-7, SKBR3, MDA-MB-231, ML-1, SCC-14C, and SCC-22A cells, in Dulbecco's modified Eagle's medium (DMEM; Biochrom KG, Berlin, Germany). These media were supplemented with 1% L-glutamine (GlutaMax; Life Technologies, Paisley, Scotland), 10% fetal calf serum (FCS; Life Technologies), 500 U/ml penicillin and 500 μg/ml streptomycin (Life Technologies).

### Patients and healthy controls

As shown in Table 1, 45 breast cancer patients (median age, 49 years; range, 31–66 years) at different stages (20 stage IV patients with overt metastases and 25 patients with apparently localized disease: stage I, one patient; stage III, 19 patients; inflammatory cancer, 5 patients) were included at the Val d'Aurelle Centre (Montpellier), as well as 11 patients with a lymphoma as the negative control group. All breast cancer patients included in this study (from 1996 through 1998) were participating in a therapeutic trial with the agreement of the local bioethics committee, and BM aspirates were taken at the time of diagnosis of their breast cancer. Concerning the control group, the BM aspirates were taken for the diagnosis of their lymphoma. Written informed consent was obtained from all patients. All 25 breast cancer patients with apparently localized disease were included after surgery but before any systemic treatment (e.g., radiotherapy/chemotherapy). BM aspiration in the 20 patients with metastatic cancer was performed before and after initiation of systemic therapy (9 and 11 patients, respectively).

**Table 1:** Patient characteristics

Variable	Patients (n = 45) Number (%)
Age	
<50 years	25 (55.6)
≥50 years	20 (44.4)
	
Tumor status	
pT_1_	9 (20)
pT_2_	19 (42.2)
pT_3_	5 (11.1)
pT_4_	11 (24.5)
pT_x_	1 (2.2)
	
Lymph node status	
pN_0_	4 (8.9)
pN_1_	41 (91.1)
	
Metastasis	
M_0_	20 (44.4)
M_1_	25 (55.6)
	
Grade	
1	2 (4.4)
2	17 (37.8)
3	23 (51.1)
Unknown	3 (6.7)
	
Estrogen-receptor and progesterone status	
ER^+^	25 (56.8)
ER^-^	19 (43.2)
PR^+^	21 (47.7)
PR^-^	23 (52.3)

### Preparation of BM

BM was aspirated from the upper iliac crest, and mononuclear cells were isolated with Ficoll-density centrifugation, as described previously [12]. Cells derived from BM samples were then cryopreserved in liquid nitrogen before the CK19-EPISPOT.

### CYFR 21-1 measurement

CYFRA 21-1 was measured with the CYFRA 21-1 immunometric assay (B.R.A.H.M.S., Hennigsdorf, Germany) in culture supernatants (Tables 2 and 3).

**Table 2:** Detection of CK19 in cancer cell lines by different methods

	Colorectal cells			Breast cells	Head/Neck cells		Thyroid cells	
				
	HT29	HCT116	Caco-2	MCF7	14C	22A	ML1	C643
Detection of intracellular CK19								
FC	+	+	+	+	-	-	-	-
ICC	+	+	+	+	-	-	-	-
WB	ND	ND	ND	+	-	-	ND	-

Detection of released CK19								
EPISPOT	+	+	+	+	-	-	-	-
ELISA	+	+	+	+	-	-	-	-

CK19 detection. FC = flow cytometry; ICC = immunocytochemistry; WB = Western blot; ND = not determined; EPISPOT = EPithelial ImmunoSPOT assay; ELISA = CYFRA 21-1 immunometric assay (B.R.A.H.M.S.).

**Table 3:** Comparative sensitivity of two immunometric assays for detection of released CK19 in cancer cells

Cells/well	HT-29 cells	HCT 116 cells	Caco-2 cells	MCF-7 cells
EPISPOT ASSAY				
1,000	212.5 ± 32.7 (21.3%)	867.0 ± 28.4 (86.7%)	600.0 ± 56.5 (60.0%)	113.3 ± 4.7 (11.4%)
100	19.8 ± 3.3 (19.8%)	93.0 ± 3.0 (93.0%)	67.5 ± 7.0 (67.5%)	13.5 ± 2.1 (13.5%)
10	2.0 ± 0 (20.0%)	7.3 ± 1.8 (73.0%)	5.5 ± 0.5 (55.0%)	2.0 ± 0.7 (20.0%)
1	0.25 ± 0.38 (25.0%)	1.0 ± 0 (100.0%)	0.4 ± 0.5 (40.0%)	0.23 ± 0.94 (23.0%)
ELISA				
1,000	2.1 ± 3.0	12.0 ± 9.2	16.7 ± 2.9	16.2 ± 5.2
100	0.2 ± 0.2	1.0 ± 1.4	1.5 ± 1.8	2.6 ± 0.7
10	0.0 ± 0.0	0.0 ± 0.0	0.0 ± 0.0	0.0 ± 0
1	0.0 ± 0.0	0.0 ± 0.0	0.0 ± 0.0	0.0 ± 0

EPISPOT and ELISA assays. Cells were cultured for 24 hours on nitrocellulose membranes of the Elispot plates. For the EPISPOT, values are expressed asCK19-releasing cells per well (mean ± SD of three experiments, with two wells in each experiment). Supernatants of the cultured cells were tested by the CYFRA 21-1 immunometric assay, and results are expressed as nanograms per milliliter.

### EPISPOT assays

The EPISPOT was performed as previously described [13]. For the CK19-EPISPOT, the anti-CK19 Ks19.1 (6 μg/ml) and the Alexa^488^-conjugated anti-CK19 Ks19.2 (3 μg/ml) mAbs (Progen Biotechnik GMBH, Heidelberg, PA) and/or and the Alexa^555^-conjugated anti-CK19 mAb AE1 (3 μg/ml) (Chemicon International, Temecula, CA) were used.

For the dual-fluorescent CK19/MUC1-EPISPOT, a mixture of anti-CK19 mAb Ks19.1 and anti-MUC1 mAb 115D8 (5 μg/ml) (Centocor, Malvern, PA) with a mixture of Alexa^488^-conjugated anti-CK19 mAb Ks19.2 and Alexa^555^-conjugated anti-MUC1 mAb DF3 (1:3,000) (Centocor) were used.

CK19-EPISPOT with Brefeldin A was performed to study the CK19 intracellular transport. Brefeldin A (3 μg/ml) was added in the culture medium of MCF-7 cells during the 24-hour cell-incubation step of the EPISPOT.

### Flow-cytometry experiments

The expression of CK19 in cancer cell lines (HT-29, HCT-116, CaCo2, MCF-7, SKBR3, MDA-MB-231, SSC-14C, SSC-22A, ML1, and C643) was determined with flow cytometry (FC 500 apparatus; Beckman-Coulter, Villepinte, France) (Table 2). Intracytoplasmic CK19 staining was performed by using the Alexa^488^- or Alexa^555^-conjugated anti-human CK19-Ks19.2 mAb (Progen Biotechnik), and the IntraPrep permeabilization reagent kit (Beckman-Coulter).

### Immunocytochemistry

Cell lines were immunostained with Alexa^488^-conjugated anti-CK19 mAb, as described earlier for the flow cytometry (Table 2). Then cells were seeded on glass slides, which were mounted in ProLong Gold antifade reagent with DAPI (Invitrogen) and analyzed (Axio Imager M1, Carl Zeiss Vision, Halbermoos, Germany).

MCF-7 cells were incubated with vincristine, 20 μmol/l (Sigma-Aldrich, Steinheim, Germany), an inducer of apoptosis, and immunostained by using the M30 CytoDEATH Fluorescein (Peviva, Bromma, Sweden) and the Alexa^555^-conjugated anti-CK19 mAb Ks19.2.

A dual immunostaining was performed on MCF-7 cells by using the Alexa^488^-conjugated anti-CK19 mAb Ks19.2 and the Alexa^555^-conjugated anti-MUC1 mAb DF3. Nuclear counterstaining was performed with DAPI.

### Western blot

To detect the expression of CK19 protein in the cell lines (MCF-7, SSC-14C, SSC-12A, and C643; Table 2), cells were lysed in Triton-DOC lysis buffer. After centrifugation, supernatants were collected and analyzed with Western blotting. Samples were mixed with an equal volume of 2 × Laemmli buffer, boiled (5 min), and then loaded onto a 12% polyacrylamide gel. Proteins were electroblotted onto Immobilon membranes, and CK19 was detected by using the Alexa^488^-conjugated anti-CK19 KS 19.2 mAb. The Immobilon membrane was scanned by using a Typhoon model 8600 phosphorimager with ImageQuant software (Molecular Dynamics, Sunnyvale, CA).

### Mass spectrometry and protein identification

MCF-7 and C643 cells were cultured in FCS-free medium and tested with the EPISPOT to confirm that full-length CK19 still could be detected. FCS-free medium was used as negative control. Five milliliters of each cell-culture supernatant was concentrated by using 5-kDa cut-off centrifugal filter units (Millipore). The concentrated supernatants were diluted with 50 mmol/l ammonium bicarbonate in a volume of 50 μl and digested with 1 μg trypsin at 37°C. Hydrophobic proteins attached to the centrifugal filter unit were digested with 1 μg trypsin in 50 μl 10% (vol/vol) acetonitril in 50 mmol/l ammonium bicarbonate at 37°C. Samples were cleaned of salts by using STAGE tips [14].

Nanoscale LC-MS/MS was performed by coupling an Agilent 1100 Series LC system to a LTQ XL quadrupole ion-trap mass spectrometer (Finnigan, San Jose, CA), as described earlier [15].

### Statistical analysis

Kaplan-Meier life-table curves were used to visualize for overall survival. The log-rank test was used to compare significance of differences between the curves.

Cox's proportional hazards regression model with a stepwise selection procedure was applied to investigate main prognostic factors. Hazards ratios with 95% CIs were presented to display modifications in the risk of death.

All statistical tests were two-sided, and a *P *value of less than 0.05 was considered statistically significant. Statistical analyses were performed by using the SAS v9 software (SAS Institute, Cary, NC).

## Results

### Release of CK19 by cancer cells

**Figure 1:**
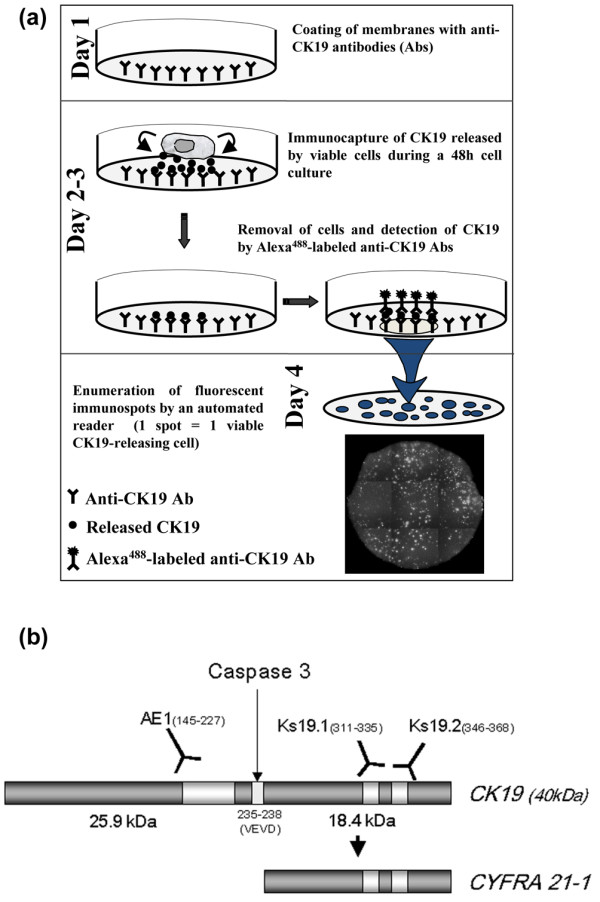
Detection of CK19 protein. **(a) **CK19-EPISPOT assay procedure. Day 1: The membranes of the ELISPOT plates are coated with an anti-human CK19 monoclonal antibody (mAb, Ks19.1). Days 2 to 3: Next, the cells are seeded in each well and cultured for 48 hours. During this incubation period, the released specific proteins are directly immunocaptured by the immobilized mAb on the bottom of the well. Plates are then washed, and cells are removed. The presence of the released CK19 protein is revealed by the addition of an anti-human CK19 mAb (Ks19.2) conjugated with a fluorochrome. Day 4: Fluorescent immunospots are counted with an automated reader. One immunospot corresponds to the fingerprint left only by one viable releasing epithelial tumor cell. **(b) **Schematic representation of the assumed binding sites of anti-cytokeratin antibodies and cleavage site for caspase 3 on the human CK19 protein [3,40].

To evaluate whether epithelial tumor cells can release the CK19 protein, we applied two immunometric assays (ELISA and EPISPOT) to a panel of different cancer cell lines (Table 2). The ELISA detected an epitope on the CYFRA 21-1 part of CK19 in the cell-culture supernatants, whereas the CK19^(Alexa488)^-EPISPOT (Figure 1a), an adaptation of the Elispot technique, detects fingerprints of the CK19 protein secreted by single epithelial cells on a solid membrane [13]. Green fluorescent immunospots correspond to viable CK19-releasing cells (RCs).

For the CK19-EPISPOT, two mouse monoclonal anti-CK19 antibodies, clones Ks19.1 (specific for the amino acids 311 to 335) and Ks19.2 (specific for the amino acids 346 to 368), recognizing both epitopes on the CYFRA 21-1 fragment, were used (Figure 1b). The antibodies used in both immunometric assays were not able to distinguish the soluble fragment CYFRA 21-1 and the full length of CK19. In parallel, we assessed the intracellular expression of CK19 protein with flow cytometry, immunocytochemistry (ICC), and Western blot experiments. CK19 expression and release were detected in colon and breast cancer cell lines but not in the head and neck squamous and thyroid cancer cell lines analyzed (Table 2). The lack of CK19 expression in the two head and neck cancer cell lines is of interest, given the reported impact of CYFRA 21-1 measurements in this disease [16]. Thus, it can be speculated that CK fragments found in the blood of head and neck cancer patients might not be the results of active release but rather reflect apoptotic or necrotic processes.

We determined the sensitivity of the CK19-EPISPOT in comparison to the CYFRA 21-1 ELISA by using the same antibody clones in both immunometric assays. Serial dilutions of the HT-29, HCT 116, Caco-2, and MCF-7 cell lines were tested (Table 3). CK19-EPISPOT was able to detect tumor cells even at the lowest concentration (1 cell/well), whereas at least 100 cells were needed to obtain a positive ELISA signal, indicating a two-orders-of-magnitude greater sensitivity of the EPISPOT. Taken together, these results indicate that the CK19-EPISPOT permitted the enumeration of very small numbers of CK19-RCs.

### Release of the full-length CK19 protein by cancer cells

Several groups demonstrated that the release of CYFRA 21-1 occurs when CK19 is cleaved by caspase 3 during apoptosis [3,17]. However, it is still debated whether MCF-7 cells expresses caspase 3 [18]. Other caspases (e.g., caspase 7 and 9) can cleave cytokeratins in a similar manner, and these caspases might be active in MCF-7 cells lacking caspase 3 [18].

**Figure 2:**
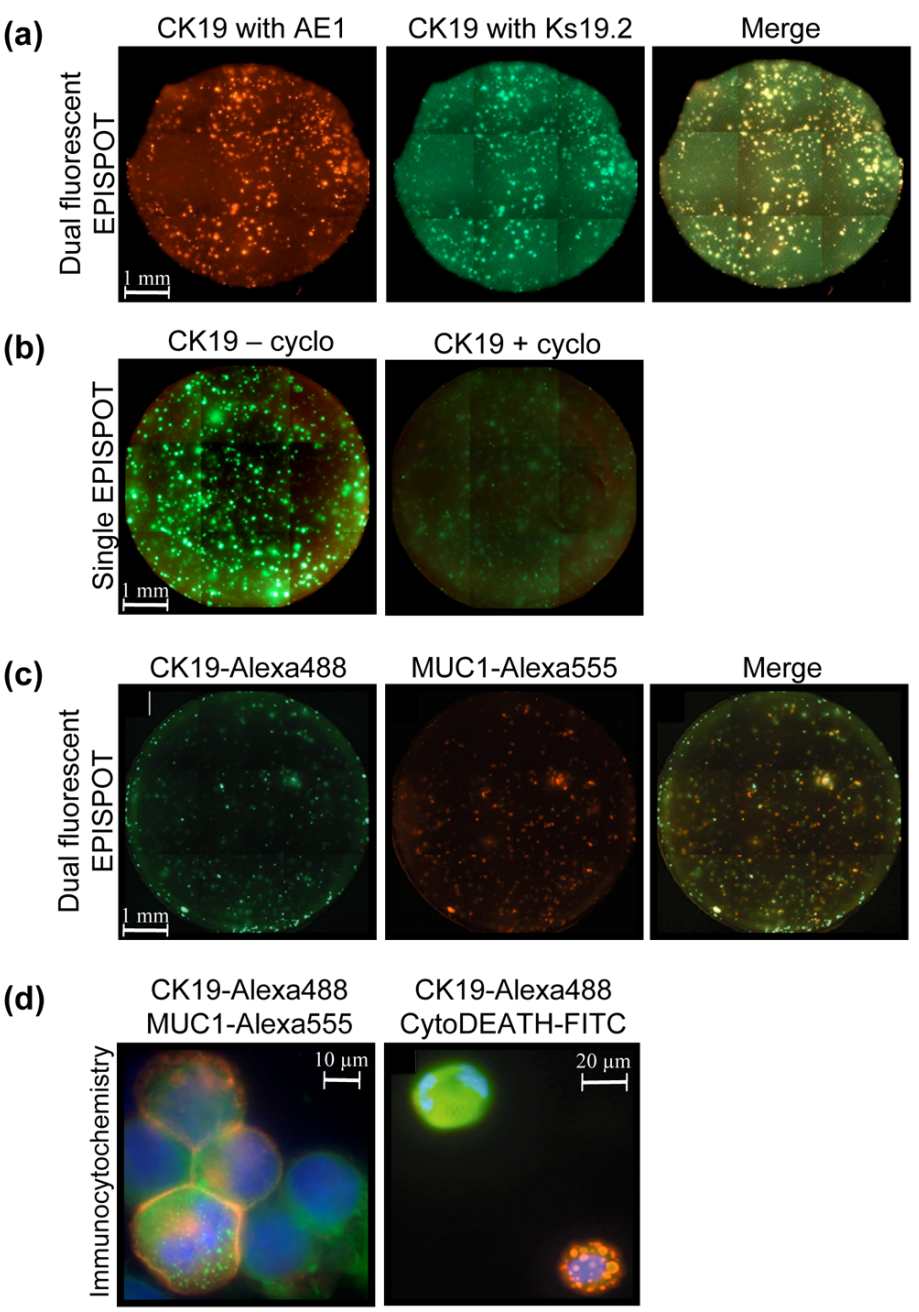
EPISPOT assays and immunocytochemistry experiments. **(a) **Dual-fluorescent CK19-EPISPOT assay with two different couples of mAbs. Red and green immunospots represent protein fingerprints of cells releasing CK19 detected with anti-CK19 AE1^Alexa555 ^and Ks19.2^Alexa488 ^mAbs, respectively. Yellow fluorescent immunospots (Merge) are the results of dual staining with both mAbs. **(b) **Single-fluorescent CK19 Ks19.2^(Alexa488)^-EPISPOT assay on MCF-7 cells without and with the addition of cycloheximide (50 μg/ml). **(c) **Dual-fluorescent CK19 Ks19.2^(Alexa488)^/MUC1^(Alexa555) ^EPISPOT assay on the MCF-7 cancer cell line. Green and red immunospots represent CK19-RCs and MUC1-RCs, respectively. Yellow fluorescent immunospots (Merge) are the results of dual staining with both mAbs. **(d) **Immunostaining of the nucleus^(dapi)^, the MUC1^(Alexa555)^, and the CK19 Ks19.2^(Alexa488) ^of breast MCF-7 cells (*left*). Immunostaining of the nucleus^(dapi)^, the CK19 Ks19.2^(Alexa555)^, and the cleaved cytokeratin 18 with the M30 CytoDEATH^(FITC)^of MCF-7 cells after an incubation with vincristine (20 μmol/l), an inducer of apoptosis (*right*). The antibody M30 CytoDEATH which recognizes a neo-epitope formed after caspase cleavage of cytokeratin 18 at Asp396 (CK18Asp396-NE M30 neo-epitope) during and after apoptosis, does not bind native CK18 of normal cells and is a very reliable and convenient tool for demonstration of apoptosis in single cells [41]. CK19 vesicles were detectable in the viable but not in the apoptotic MCF-7 cells.

To demonstrate that the CK19 protein released by the cancer cells analyzed was the intact full-length CK19 protein and not its soluble fragment, we developed a dual-fluorescent CK19-EPISPOT that is able to distinguish both peptides (i.e., the full length CK19 and the CYFRA 21-1). Thus, for the coating step, the same anti-CK19 antibody Ks19.1 that recognizes an epitope on the CYFRA 21-1 fragment of the CK19 (amino acids 311 to 335) (Figure 1b) was used. For the revelation step, we used monoclonal antibody Ks19.2 recognizing CK19 at the amino acids 346 to 368 and monoclonal antibody AE1 recognizing CK19 on a different epitope, localized on the other side of the cut site of the caspase 3 at the amino acids 145 to 227 (Figure 1b). Ks19.2 and AE1 were conjugated with two different fluorochromes (i.e., Alexa^488 ^and Alexa^555^, respectively). By using this dual-fluorescent CK19^(Alexa488)^/CK19^(Alexa555)^-EPISPOT on MCF-7 breast cancer cells only dual^(yellow) ^immunospots were observed (Figure 2a), indicating that all cells released the intact full-length CK19 protein. Similar results were obtained with the two other breast cancer cell lines, SKBR3 and MDA-MB-231 (data not shown).

In addition, the release of full-length CK19 protein (400 amino acids) by MCF-7 cells was investigated with peptide sequencing by using nanoscale reversed-phase liquid chromatography tandem mass spectrometry (LC MS/MS analysis) (data not shown). Peptides were detected ranging from CK19 amino acids 8 to 398, which demonstrated that the full-length CK19 was present in the cell-free supernatant. Importantly, the tryptic peptide 'GQVGGQVSVEVDSAPGTDLAK' (amino acids 227 to 247), which can emerge only from the entire CK19 protein, was detected (data not shown).

### Active release of CK19 by viable cancer cells

It has been reported that the CK19 is released by apoptotic or necrotic cells [19,20]. We reversely inhibited translation by incubation of the cells with cycloheximide to analyze whether CK19 release is an active process that requires protein translation. The addition of cycloheximide to cell cultures before and during the incubation step of the CK19-EPISPOT led to a decrease in the size and the number of CK19 spots or even their disappearance, confirming that the *de novo *synthesis of the CK19 protein by viable epithelial cancer cells is required for the active release of CK19 (Figure 2b). Moreover, to study the CK19-release pathway, we inhibited the protein transport in the Golgi apparatus by incubation of the cells with the lactone antibiotic, Brefeldin A. The addition of Brefeldin A to cell culture during the CK19-EPISPOT did not affect the CK19 release, demonstrating that the CK19 protein is not in transit through the endoplasmic reticulum or the Golgi apparatus (data not shown).

MUC1 is another specific tumor-associated marker that has been already used for the detection of disseminating tumor cells [21,22]. We previously showed that the release of MUC1 is restricted to viable tumor cells (e.g., MCF-7) [13]. We therefore optimized a dual-fluorescent MUC1^(Alexa555)^/CK19^(Alexa488)^-EPISPOT to verify whether these cells were able to release both proteins simultaneously. We observed that a majority of the immunospots obtained after 24 hours of cell culture were yellow, resulting from an equivalent mixture of the released MUC1 and CK19 proteins and confirming that these cells were viable cells able to neosynthetize and actively release *in vitro *MUC1 and CK19 (Figure 2c). In additional ICC experiments, we showed that MCF-7 cells with an intact nucleus (DAPI staining) contained CK19 vesicles with a peripheral MUC1 expression at the cell membrane (Figure 2d). Induction of apoptosis with vincristine and green staining of apoptotic cells showed that these CK19 vesicles were detectable only in the subset of nonapoptotic MCF-7 cells (Figure 2d).

### CK19-releasing cells in BM of breast cancer patients

The detection and characterization of DTCs in the BM of patients with solid epithelial tumors is an emerging clinical research field, in particular in breast cancer patients [23]. Recently, we demonstrated the presence of CK19-RCs, even in the absence of overt metastases (stage M_0_), indicating clinically occult homing of viable cancer cells in BM [11]. Here, we confirmed the specificity of the CK19-EPISPOT by demonstrating consistently negative findings in 11 BM samples from control patients (data not shown).

The main purpose of the present study was to obtain information on the prognostic relevance of CK19-RCs. Breast cancer patients were followed up for a median period of 6 years, and comparison of survival distribution with Kaplan-Meier analysis by using the log-rank test was performed. The clinical information on the patients is shown in Table 1. Most patients were in an advanced disease stage. Of the 25 M_0_-patients (55.5%), 6 patients were free of lymph-node metastases, whereas the remaining 19 patients already had histologically detectable nodal metastasis. Twenty (44.5%) M_1 _patients had overt distant metastases localized in BM/liver (n = 6), BM (n = 3), lung (n = 3), liver (n = 2), BM/lung (n = 2), lung/liver (n = 1), liver/brain (n = 1), skin (n = 1), and BM/liver/lung (n = 1). Among 45 breast cancer patients, 19 experienced disease recurrence: local relapses were diagnosed in two (4.5%) patients, and new distant metastases were diagnosed in 17 (38%) patients in the liver (n = 4), BM (n = 4), lung (n = 3), BM/brain (n = 2), mediastinum (n = 1), skin/lung (n = 1), skin (n = 1), bone/meningitis (n = 1), and pleura (n = 1). Twenty-nine (64.5%) deaths were reported.

All M_0 _patients received an anthracycline-based chemotherapy regimen. Considering the bad prognosis of the patients with massive lymph node involvement, 8 patients received, in addition, a high dose of chemotherapy with autologous bone marrow transplantation, and 5 patients received a sequential association of anthracycline and docetaxel. Nearly all patients (except for two) with hormone receptor-positive tumors received adjuvant tamoxifen therapy.

All M_1 _patients received a chemotherapeutic treatment after the bone marrow sampling. Considering the heterogeneity of settings of this population, the numerous chemotherapeutic regimens (e.g., four cycles of the FAC65 regimen, seven cycles of a sequential association AC65-docetaxel, four cycles of an FEC100 regimen, and then autologous bone marrow transplantation) are not reported here in detail.

The enumeration CK19-RCs and MUC1-RCs allowed the detection of viable DTC in 44% and 48% of M_0 _patients, respectively, whereas the corresponding detection rates were 70% and 65% in M_1 _patients, respectively. If the results of both CK19- and MUC1-RC measurements were combined, it showed that viable DTCs were present in 56% and 90% of M_0 _patients and M_1 _patients, respectively. The number of DTCs was significantly higher (*P *= 0.01) in the M_1 _patients (CK19-RCs: median, 7; range, 0 to 758; MUC1-RCs: median, 10; range, 0 to 812) compared with the M_0 _patients (CK19-RCs: median, 0; range, 0 to 254; MUC1-RCs: median, 0; range, 0 to 32).

**Figure 3:**
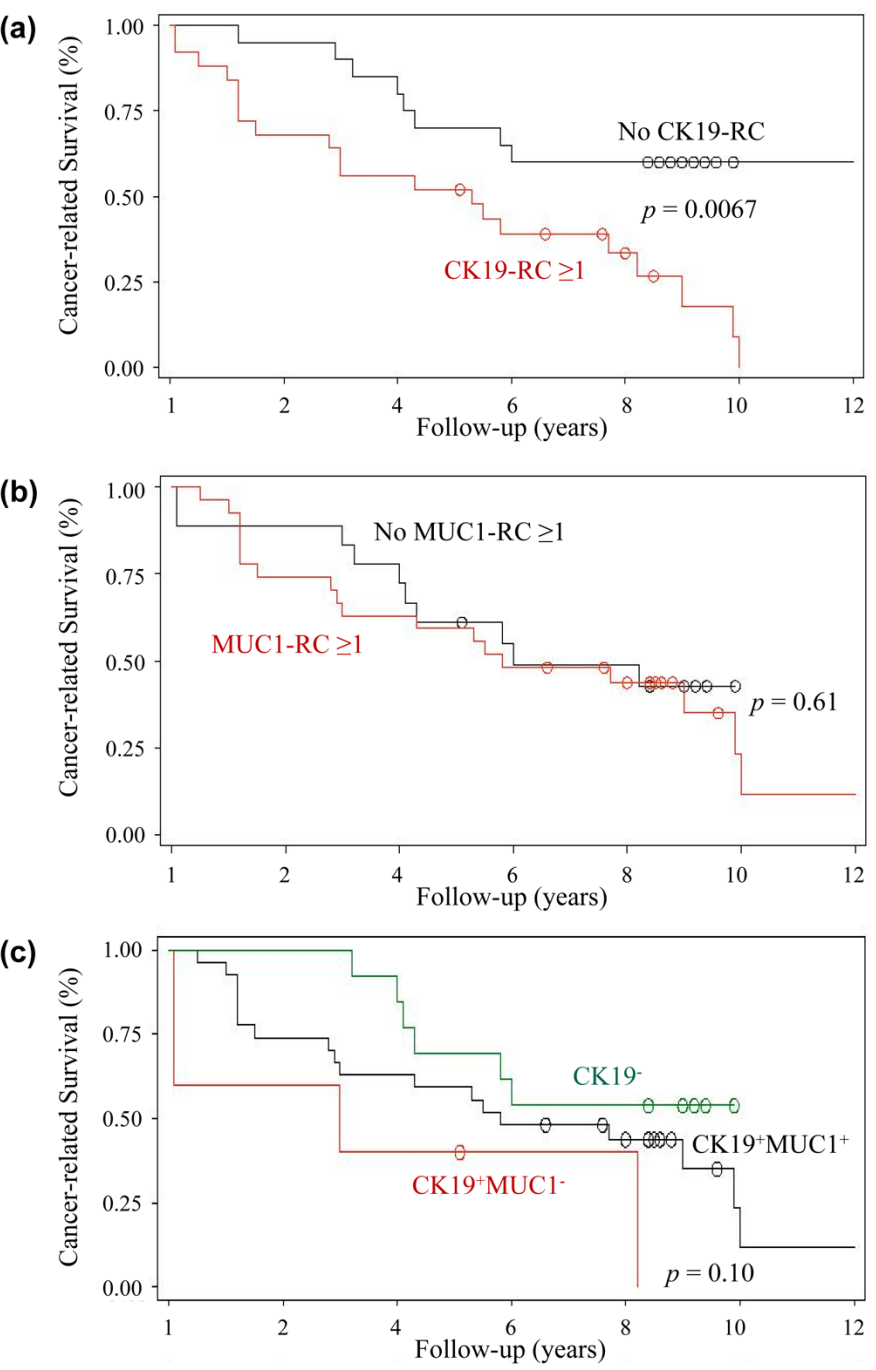
Survival data. Kaplan – Meier estimates of cancer-related survival of breast cancer patients, according to the presence or absence of viable CK19-RCs. **(a)**, MUC1-RCs **(b)**, and subpopulations of cells CK19^+^MUC1^-^, CK19^+^MUC1^+ ^**(c) **in the BM. RCs = releasing cells.

We calculated the Kaplan-Meier curves for patients, depending on the presence or absence of either CK19-RCs or MUC1-RCs. As shown in Figure 3a, the detection of CK19-RC was prognostically relevant (*P *= 0.0067); no obvious difference was noted in the sites of metastasis between patients with and without CK19-RCs. In contrast, the detection of MUC1-RCs had no prognostic impact (*P *= 0.6106; Figure 3b).

We also calculated the Kaplan-Meier curves for the three subsets CK19^-^, CK19^+^MUC1^+^, and CK19^+^MUC1^- ^(Figure 3c). Because of the low number of cases, the *P *value (*P *= 0.1027) did not reach statistical significance, but the results showed that breast cancer patients harboring the subset of CK19^+^MUC1^- ^DTC had the worse survival curve.

Subsequently, multivariate analysis was performed, taking the most important variables into account. Type of therapy was strongly linked to TNM stage and was therefore not considered an independent variable. As shown in Table 4, the presence of CK19-RCs was the strongest prognostic predictor, with a hazard ratio of 4.7 and a *P *value of 0.0019. Because of the limited number of patients, the nature of the multivariate is, however, rather exploratory.

**Table 4:** Multivariate hazard ratios for death from breast cancer

Variable	Hazard ratio (95% CI)	*P *value
CK19-RC	4.71 (1.78–12.52)	0.0019
MUC1-RC	0.44 (0.18–1.07)	0.0710
ER status	0.45 (0.20–1.01)	0.0528
TNM stage	1.92 (0.92–3.99)	0.0830

CI = confidence interval; RC = releasing cell; ER = estrogen receptor.

## Discussion

In contrast to the common concept that cytokeratins are released in fragmented forms only by epithelial cells (e.g., as a result of apoptosis), we provide here the first evidence for a release of full-length CK19 by viable epithelial tumor cells. Control experiments were performed to demonstrate that this release was an active process and not simply the consequence of apoptosis. In addition, our clinical follow-up results – although still preliminary – suggest that viable tumor cells secreting CK19 may contribute to metastatic progression.

By using the EPISPOT on different breast and colon cancer cell lines with monoclonal antibodies targeting different epitopes of CK19, we showed that a significant fraction of these cells release the intact full-length CK19. As immunospots are the protein fingerprints left only by the viable RCs, a cell culture is needed to accumulate a sufficient amount of the released marker proteins. Thus, dying cells that do not secrete adequate amounts of protein are not detected, as previously described [13,24,25]. The full-length nature of the released CK19 protein was confirmed by proteomic experiments on MCF-7 breast cancer cell supernatants: (a) we did not detect a specific tryptic fragment peptide, which should have been detected if the CYFRA 21-1 were present in the culture supernatant; and (b) we found a specific hydrophobic peptide (amino acids 227 to 247), which can emerge only from the intact insoluble CK19 protein.

By using different combinations of dual-fluorescent EPISPOT, immunocytochemical analyses and cycloheximide, Brefeldin A or vincristine cell-incubation, we obtained the first evidence that the release of the intact full-length CK19 protein might be an active process, probably mediated by vacuoles or vesicles but using another protein-transport pathway than that between the classic endoplasmic reticulum to the Golgi apparatus. The precise mechanism, however, remains to be elucidated. Keratin-like antigens can be released during cell invasion [26], and ultrastructural studies indicated an association of keratin proteins with secretory granules [27] and mucin droplets [28]. However, the CK19 molecule lacks a secretory leader and hydrophobic membrane-translocation sequence. Moreover, it has no precursor [29], and it has never been located in the endoplasmic reticulum and Golgi apparatus. A possible mechanism might be blebbing of proteins of the cytoplasm surrounded by plasma membrane (like viral budding [30]) or the release through exosomes known to contain cytoskeletal proteins and to be involved in metastasis [31].

In a first attempt to unravel whether the release of CK19 has a biologic function in tumor progression, we applied the CK19-EPISPOT to detect with a high sensitivity viable cells releasing CK19 in the BM of breast cancer patients. The consistent negative findings in samples from patients without carcinoma supported the assumption that CK19-RCs appear to be DTCs. CK19-RCs were frequently found in the BM of breast cancer patients, and the number of CK19-RCs was correlated with the disease stage. This observation is consistent with the fact that BM is the most prominent distant site for metastatic progression in breast cancer [7]. Most important, our clinical follow-up data suggest, for the first time, that the release of CK19 seems to be important for the occurrence (M_0 _patients) or progression (M_1 _patients) of metastasis in breast cancer patients. Although the biologic role of CK19 release remains to be elucidated, it might be noteworthy that another cytokeratin (keratin 17) was recently implicated in epithelial cell growth regulation [32]. Moreover, Ding *et al*. [33] showed that overexpression of CK19 in hepatocellular carcinoma cells is related to metastatic behavior.

The assumption that not all DTCs are relevant to metastatic progression was emphasized by the present observation that, in contrast to CK19-RCs, DTCs releasing MUC1 had no prognostic impact. However, the number of patients analyzed in our study is far too small to exclude that MUC1-expressing cells might have clinical relevance in certain subsets of breast cancer [34]. Nevertheless, our results indicate that DTCs with different phenotypes appear to have a heterogeneous potential to contribute to metastatic relapse, which is consistent with previous reports on the expression of the HER2 oncogene [35] and the urokinase-type plasminogen-activator receptor [36,37] on DTCs.

Although we cannot exclude the possibility that CK19 release is only a marker for the detection of DTCs, we propose that DTCs with the CK19^+^/MUC1^- ^phenotype might have a particular biologic potential. This assumption also is supported by the findings of Gudjonsson *et al*. [8], who reported that CK19^+^/MUC1^- ^cells in the human breast may have stem cell-like properties. These data are consistent with a report from Max Wicha's group [9] on mouse mammary cells, which showed that CK19 might be a putative stem-cell marker and the findings of Peterson *et al*. [10] indicating CK19 as a marker of breast cancer progenitors.

## Conclusions

In conclusion, our present data suggest a potential role of CK19 release as an indicator of aggressive behavior of disseminated breast cancer cells. Besides further investigation of the underlying mechanism, larger studies are now required to evaluate whether the CK19-EPISPOT will provide more helpful clinical information than the current immunocytochemical or RT-PCR-based DTC detection assays [38]. In addition, the EPISPOT can be applied to the detection and characterization of circulating tumor cells in the peripheral blood, an application that has generated substantial interest over the past years [23,39].

## Abbreviations

BM: bone marrow; CK: cytokeratin; DTCs: disseminated tumor cells; EPISPOT: epithelial immunospot; FC: flow cytometry; FCS: fetal calf serum; ICC: immunocytochemistry; mAb: monoclonal antibody; RCs: releasing cells; WB: Western blot.

## Competing interests

The authors declare that they have no competing interests.

## Authors' contributions

CAP and KP carried out the study design, data mining, data analysis, and manuscript writing. JPV helped to draft the manuscript. MS carried out the proteomic experiments. CAP and OP carried out the research, in particular, the EPISPOT assays. EB and GM carried out the statistical analyses. MF carried out the inclusions of the breast cancer patients, and WJ helped to gather clinical data for all the patients. All authors read and approved the final manuscript.
